# Genomic epidemiology and resistome dynamics of *Enterobacter* species in a Portuguese Open Air Laboratory: the emergence of the FRI-8 carbapenemase

**DOI:** 10.3389/fmicb.2025.1593872

**Published:** 2025-07-31

**Authors:** Pedro Teixeira, Miguel Ramos, Rani Rivière, Mónica Azevedo, Mário Ferreira, Maria Manuela Cano, Patrícia Vieira, Lígia Reis, Rui Matias, João Rodrigues, Carina Menezes, Tânia Rosado, António Sequeira, Olga Moreira, Werner Ruppitsch, Adriana Cabal-Rosel, Solveig Sølverød Mo, Elsa Dias, Markus Woegerbauer, Manuela Caniça, Vera Manageiro

**Affiliations:** ^1^National Reference Laboratory of Antibiotic Resistances and Healthcare Associated Infections, Department of Infectious Diseases, National Institute of Health Dr. Ricardo Jorge (INSA), Lisbon, Portugal; ^2^Centre for the Studies of Animal Science, Institute of Agrarian and Agri-Food Sciences and Technologies, University of Porto, Porto, Portugal; ^3^AL4AnimalS, Associate Laboratory for Animal and Veterinary Sciences, Lisbon, Portugal; ^4^Air Quality Laboratory, Department of Health Environmental Health, INSA, Lisbon, Portugal; ^5^Laboratory of Microbiology, Department of Infectious Diseases, INSA, Lisbon, Portugal; ^6^Laboratory of Biology and Ecotoxicology, Department of Environmental Health, INSA, Lisbon, Portugal; ^7^Strategic Research Unit for Animal Production and Health, National Institute of Agrarian and Veterinary Research, I.P. (INIAV), Pólo de Investigação da Fonte Boa, Lisbon, Portugal; ^8^Austrian Agency for Health and Food Safety, Department for Risk Assessment, Vienna, Austria; ^9^Faculty of Food Technology, Food Safety and Ecology, University of Donja Gorica, Podgorica, Montenegro; ^10^Department of Animal Health, Welfare and Food Safety, Norwegian Veterinary Institute, Ås, Norway; ^11^Faculty of Veterinary Medicine, CIISA, Center for Interdisciplinary Research in Animal Health, University of Lisbon, Lisbon, Portugal

**Keywords:** *Enterobacter vonholyi*, carbapenem-resistant *Enterobacterales*, colistin-resistance, FRI-8 and IMI-6 carbapenemases, MCR-10, one health

## Abstract

Interconnected reservoirs contribute to the global spread of antimicrobial resistance (AMR), including carbapenem- and colistin-resistant *Enterobacterales*, highlighting the need for a One Health approach. We assessed the genomic epidemiology, diversity and AMR mechanisms of *Enterobacter* spp. across interconnected human, animal, plant, and environmental reservoirs in a Portuguese Open Air Laboratory. Over a one year monitoring period, samples from 12 different compartments were collected and processed using selective media to isolate *Enterobacter* spp., which were subjected to antibiotic susceptibility testing, whole-genome sequencing and subsequent analyses to identify AMR determinants, characterize plasmids and phylogenetic relationships. We established a collection of 61 *Enterobacter* isolates spanning nine species and 32 sequence types, including 16 novel ones, across nine compartments (river water, wastewater, soil, manure, feed, air, farmers, pigs, wild animals), reflecting the diversity and ubiquity of *Enterobacter* species. Core-genome analysis revealed eight genetic clusters, suggesting clonal transmission across compartments. In total, 29 antibiotic resistance genes were detected across all isolates. Notably, this is the first documentation of *bla*_FRI_-harbouring *Enterobacterales* in European environmental settings and the first to describe *bla*_FRI_, *bla*_IMI_ and *mcr-10* genes in Portugal. *bla*_FRI-8_ was detected in all *E. vonholyi* isolates (*n* = 17), located on four different IncFII(Yp) plasmids, and *bla*_IMI-6_ in an *E. asburiae* isolate, flanked by IS3 family transposases. *E. vonholyi* and the *bla*_IMI-6_-harbouring *E. asburiae* isolate were resistant to carbapenems. A *mcr-10.1* gene was identified in an *E. roggenkampii* isolate on an IncFII(pECLA) plasmid. These plasmids exhibited high sequence similarity with global counterparts, indicating potential for horizontal gene transfer. Other antimicrobial resistance genes included *qnrE1*, *sul1*, and *aadA2*. Our findings underscore the importance of *Enterobacter* as vectors for AMR and the critical role of environmental compartments in its dissemination, reinforcing the importance of adopting a One Health approach to fully understand AMR dynamics.

## 1 Introduction

Antimicrobial resistance (AMR) stands among the World Health Organization’s (WHO) top 10 global health threats (UNEP, 2023). In 2019, AMR was linked to 4.95 million deaths worldwide (Murray et al., 2022). This threat extends beyond clinical settings, prompting international organizations to adopt strategic One Health approaches to combat AMR (European Commission, 2017; UNEP, 2023). The environment serves as a reservoir and driver of AMR transmission and evolution (Larsson and Flach, 2021). Within this challenge, carbapenem-resistant *Enterobacterales* (CRE) pose a major threat, classified by WHO as critical Bacterial Priority Pathogens List for activities related to surveillance and control of antibacterial resistance (World Health Organization, 2024). Moreover, CRE pervasive presence across diverse environmental compartments highlights the urgency of addressing their spread (Mills and Lee, 2019; Zhang et al., 2021). For instance, Wang and co-workers identified common NDM-positive *Escherichia coli* isolates shared among farms, flies, dogs and farmers in a Chinese poultry production, providing direct evidence of carbapenem- resistant *E. coli* transmission and environmental contamination (Wang et al., 2017). Additionally, two environmentally-sourced CRE infections which have been reported in literature, with CRE being transmitted from river water to humans (Laurens et al., 2018; Loucif et al., 2018).

The limited availability of effective antibiotic for CRE infections frequently leaves physicians to rely on older antibiotics such as colistin, despite their known toxicity (Binsker et al., 2022). The clinical efficacy of carbapenems and colistin is threatened mainly by the spread of carbapenemase-encoding plasmids and plasmid-mediated mobile colistin resistance (*mcr*) genes, respectively (Bush and Bradford, 2020; Ling et al., 2020). While the most prevalent carbapenemases (e.g., KPC, NDM, OXA-48, VIM) have long dominated discussions on AMR, lesser-known variants such as IMI and FRI, are becoming increasingly more frequent, particularly in *Enterobacter* spp. (Boyd et al., 2017; Gomi et al., 2022; Blanco-Martín et al., 2023; Emeraud et al., 2024). *bla*_FRI_ and *bla*_IMI_ genes are predominantly found within plasmid structures, along with other mobile-genetic elements (MGE), facilitating their dissemination (Boyd et al., 2017; Brouwer et al., 2019; Uwamino et al., 2019; Gomi et al., 2022; Blanco-Martín et al., 2023).

*Enterobacter* spp. are part of the ESKAPE pathogens; overall, they are of paramount importance due to their capacity to acquire antibiotic-resistance genes (ARGs), which reduce the treatment options of serious infections (Rice, 2008; De Oliveira et al., 2020). Reports describing the prevalence of *Enterobacter* isolates harboring *mcr* and/or carbapenemase-encoding genes in both clinical (Hendrickx et al., 2021; Liao et al., 2022; Li et al., 2023) and environmental settings (Roschanski et al., 2019; Manageiro et al., 2020, 2022; Xu et al., 2021), underscore their serious threat to human health.

While the relevance of the environment in the context of AMR dissemination is well recognized, unraveling the complexities of the involved transmission networks and development mechanisms requires a multi-dimensional approach (Larsson et al., 2018; Larsson and Flach, 2021). Therefore, this study aimed to fill knowledge gaps in the genomic epidemiology and AMR mechanisms of *Enterobacter* spp. isolates collected within a Portuguese Open Air Laboratory (OAL) using Whole-Genome Sequencing (WGS). By using this real experimental research facility, the study allowed to evaluate the dynamics of the environmental resistome and bacterial diversity across different, yet interconnected compartments.

## 2 Materials and methods

### 2.1 Study sites and sample collection

**Figure 1:**
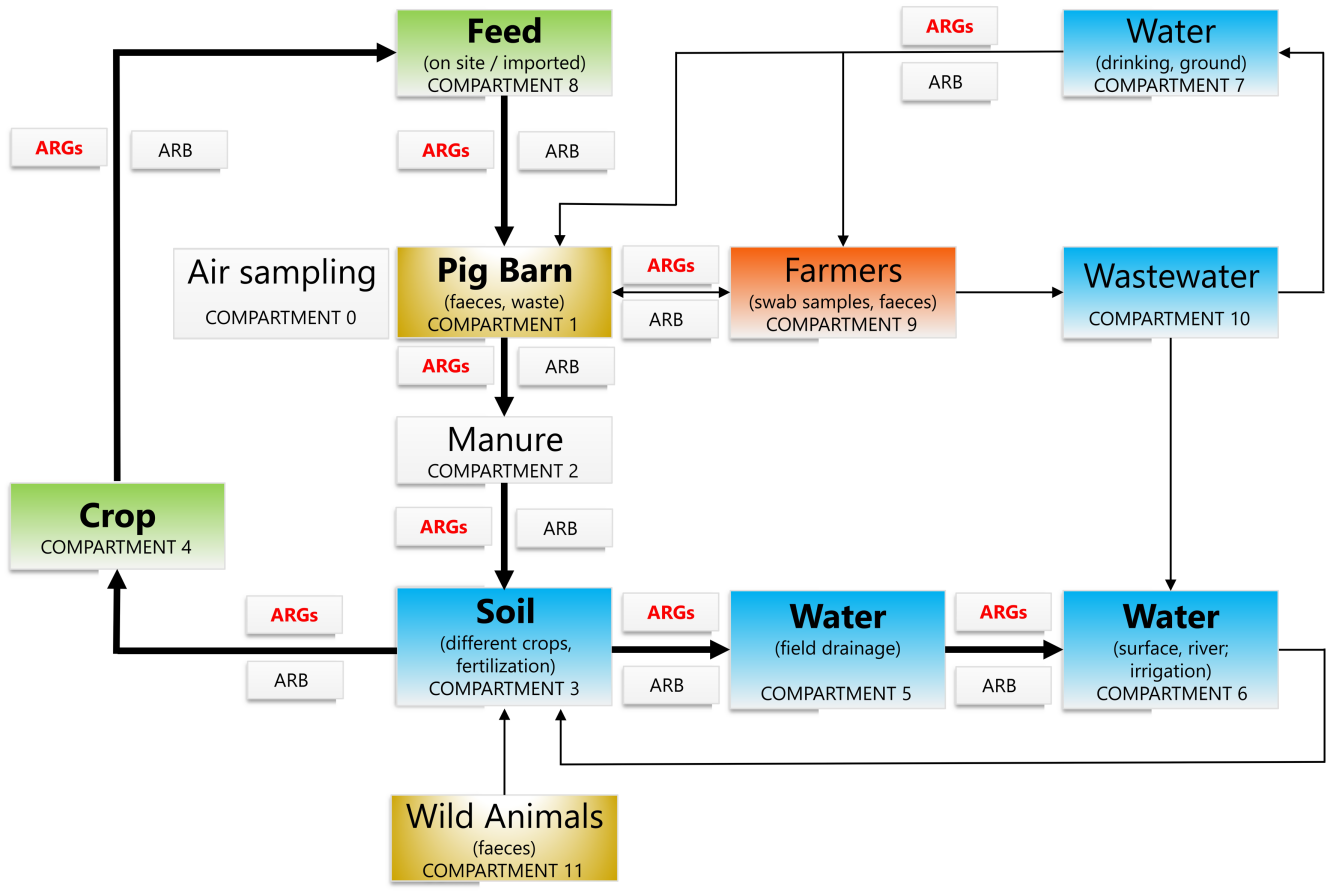
Schematic view of all the compartments from which samples were collected and the environmental ecosystem barriers through which ARG dissemination can occur. Bold letters/arrows = compartments and pathways for ARG movement that are monitored on the OAL testing range. ARGs: antimicrobial resistance genes, ARBs: antimicrobial resistant bacteria. Animal compartments (pigs at stable; wild animals in OAL catchment area): gold. Human compartments (workers exposed to animal husbandry in OAL): red. Compartments associated to plants (crops, animal feed): green. Genuine environmental compartments (soil, water): blue. Other environmental compartments (air sampling, manure): white.

Samples were collected from July 2020 to May 2021 (4 seasons), from 12 different compartments (Figure 1), at an experimental agricultural and agri-food production station in Portugal - the Open Air Laboratory (OAL) catchment area - which is located in Santarém (Supplementary Figure S1). This OAL is located at the Portuguese Research Station for Animal Production (EZN-INIAV). The catchment area is 230 ha and is divided into: 17.6% arable land, 38.2% pasture, 13.2% forested, 30.9% paved area. The wastewater treatment is performed in three waste stabilization ponds.

The samples collected for this study were obtained from the following compartments (Figure 1): C0, air of pig barns; C1, pigs from pig barns (feces); C2, manure (liquid and solid); C3, soils [soils without organic fertilization before crops cultivation, soils without organic fertilization (1 and 4 weeks) after crops cultivation; soils with organic fertilization (1 and 4 weeks) after crops cultivation; soils with or without organic fertilization before crops harvest; soils collected serving as controls: forest and meadow]; C4, crop [vetch with oats at a sowing density corresponding to 140 kg per hectare of sown land (120 kg:20 kg), obtained from manured or artificially fertilized soil]; C5, field drainage (drainage water from a borehole); C6, river (surface water); C7, drinking water (tap) and groundwater; C8, feed (on-site composed of corn, wheat, soybean bagasse, sunflower pomace, calcium carbonate, L-lysine, sea salt, wheat bran, soybean oil and vitamin supplements); C9, farmers (oral swabs and feces), C10, wastewater [effluent (prior to discharge into receiving waters), influent (wastewater from a collection tank), sedimentation tank, sewage sludge], and C11, wild animals (feces). Samples were collected at the same time each day according to the respective procedures.^1^ The initial test portion of soil or feces, manure, solid feed, and water were mixed with buffered peptone water and subsequently seeded on selective and differential medium, MacConkey and/or UriSelect™-4-agar (BioRad, France), with and without cefotaxime 2 mg/L or colistin 0.5 mg/L, to select Gram negative bacteria.^2^ Oral swabs from farmers were plated on the same culture medium and antibiotics, but also on chocolate agar with PolyViteX™ medium (BioRad, France). Additionally, the water samples were also plated on non-selective media such as R2A (Merck, Germany) and Nutrient agar (BioKar Diagnostics, France) with and without the same antibiotics. The determination of culturable bacteria suspended in air (*n* = 63) was carried out in accordance with Standard EN13098 using MAS 100 microbiological air sampler (Merck, Darmstadt, Germany) and in polycarbonate filter, 37 mm, 0.8 μm pore size (Pall Corporation, United States). The air samplers were fixed a height of 1.5 m above the floor (EN 13098:2019^3^). Colonies with different morphology, from any of the medium above, were re-inoculated in simple agar medium (Oxoid, UK) to obtain pure cultures. Cultures were preserved at −80°C. The identification of bacterial isolates was carried out using a VITEK® MS mass spectrometer V3.2.0 (BioMérieux, Marcy-l’Étoile, France).

### 2.2 Antibiotic susceptibility testing

The phenotypic determination of the susceptibility of the 61 isolates under study was carried out by disk diffusion, against 20 antibiotics belonging to six classes: aztreonam (30 μg), amoxicillin/clavulanic acid (20 μg + 10 μg), cefotaxime (30 μg), cefepime (30 μg), imipenem (10 μg), ceftazidime (10 μg), piperacillin-tazobactam (36 μg), meropenem (10 μg), ertapenem (10 μg), cefoxitin (30 μg), ciprofloxacin (5 μg), sulfamethxazole-trimethoprim (25 μg), gentamicin (10 μg), ampicillin (10 μg), ceftiofur (30 μg), enrofloxacin (5 μg), streptomycin (10 μg) and chloramphenicol (30 μg) (BioRad, France) (Supplementary Table S2). The results obtained were interpreted according to the critical diameters defined by EUCAST v.2021 (European Committee on Antimicrobial Susceptibility Testing; EUCAST, 2021), except for the antibiotics streptomycin/tetracycline and ceftiofur/enrofloxacin whose interpretation was carried out taking into account the values defined by CLSI (2021).^4^

The determination of the minimum inhibitory concentration (MIC) for colistin was carried out using the microdilution method, with in-house 96-well broth microdilution plates prepared at the National Institute of Health Dr. Ricardo Jorge (INSA); it followed the EUCAST guidelines, as well as the “MIC testing” according to EN/ISO 17025. The strains *E. coli* ATCC 25922 and *E. coli* NCTC 13846 were used as susceptibility and resistance controls, respectively. Isolates were classified as susceptible or resistant to colistin according to the critical concentrations defined by EUCAST guidelines (2021) for *E. cloacae* (susceptible ≤2 mg/L; resistant >2 mg/L).^5^

### 2.3 WGS and *in silico* analyses

#### 2.3.1 Illumina short-read sequencing

The collection of 61 isolates identified as *Enterobacter* spp. were subjected to WGS and subsequent analysis. First, genomic DNA was extracted using the Magna Pure 96 system (Roche, Germany), in line with the manufacturer’s instructions, and quantified using Qubit™ 4 fluorometer (Thermo Scientific, United States). Sequencing libraries were prepared using a Nextera XT library preparation kit (Illumina, United States) and sequenced on an Illumina MiSeq (Illumina, United States) with 150 bp paired-end reads. Raw reads quality control and *de novo* assembly were performed using INNUca (v4.2.2).^6^ Shortly, assessment of the read’s quality and trimming was performed using FastQC (v0.11.5)^7^ and Trimmomatic (v0.38) (Bolger et al., 2014), respectively. Genomes were assembled with SPAdes (v3.14.0) (Bankevich et al., 2012) and then improved with Pilon v1.23 (Walker et al., 2014).

#### 2.3.2 Nanopore long-read sequencing and hybrid assembly

Isolates selected for plasmid assembly (*n* = 5) were subjected to long-read MinION sequencing (Oxford Nanopore Technologies, Oxford, UK) using the following criteria: isolates harboring *bla*_FRI-8_ and exhibiting plasmid sequences distinct from each other (*n* = 4) and the single *mcr-10.1* harboring isolate. DNA library was prepared using the SQK-RBK114.24 Rapid Barcoding Kit, loaded in a MinION R10.4.1 flowcell and sequenced for 20 h on an Mk1C device. Basecalling and barcode trimming was performed during the sequencing run with Guppy v7.1.4 and the Fast model options selected on the MinKNOW v23.07.12 software. Subsequently, overall read quality was inspected with pycoQC v2.5.2. and *de novo* hybrid assembly was carried using Unicycler v0.5.0 (Wick et al., 2017).

#### 2.3.3 *In silico* analysis for species identification, AMR determinants and plasmids characterization

Draft genome sequences were annotated using the automated Prokaryotic Genome Annotation Pipeline (PGAP-6.7) (Tatusova et al., 2016). Species identification was determined by calculating the average nucleotide identity (ANI) using FastANI (v1.33) against complete assembled reference genomes of *Enterobacter* spp. type strains downloaded from NCBI Genbank database,^8^ using an ANI value of 95% as cut-off (Jain et al., 2018). ARGs were screened using abriTAMR v1.0.14 and ABRicate (v1.0.1)^9^ (Sherry et al., 2023). The latter incorporates Resfinder (16.11.22) (Bortolaia et al., 2020), CARD (16.11.22) (Alcock et al., 2023), while specifically employing PlasmidFinder (16.11.22) (Carattoli et al., 2014) for the replicon typing of plasmid incompatibility groups. Furthermore, abriTAMR “plus” database was used to screen for virulence genes.

Plasmid sequences were aligned and visualized using BLAST Ring Generator (BRIG) v0.95, with the standard parameters (50% lower – 70% upper cut-off for identity and *E*-value of 10) using the pF4100 and pF821 plasmids as template for the *bla*_FRI-8_ and *mcr-10.1* comparisons, respectively (Alikhan et al., 2011). Genetic contexts of *bla*_FRI-8_, *bla*_IMI-6_ and *mcr-10.1* were analyzed with pyGenomeViz (v0.4.4).^10^ pJBIWA005_1 (CP074160) and p3442-FRI-1 (CP033467) were included for *bla*_FRI-8_ comparison, pAR_0072 (CP026851) and pEk72 (CP088230) for *mcr-10.1*, pIMI-6 (KX786187) for *bla*_IMI-6_ and pRHBSTW-00016_2 (CP058188) for class 1 integron.

#### 2.3.4 Phylogenetic analyses

*In silico* multi-locus sequence typing (MLST) prediction was performed using the PubMLST database for *E. cloacae* and new sequence types (STs) were submitted (Jolley et al., 2018). The phylogeny of *Enterobacter* spp. isolates was also evaluated using core genome MLST (cgMLST) and core genome single nucleotide polymorphism (cgSNP) analysis. The tool chewBBACA v.3.3.0^11^ was employed to create a cgMLST schema for a collection of 6,586 *Enterobacter* genomes annotated by NCBI RefSeq (619 complete genomes and 5,967 draft genome assemblies deposited on the NCBI databases, downloaded on September 18th, 2024) (Silva et al., 2018). For reference-based mapping and SNP/InDel analysis, Snippy v4.6.0^12^ was used, with the NCBI RefSeq *E. cloacae* 1,382 complete genome (NZ_OW968328.1) serving as the reference. Putative repetitive sections and recombination events were filtered using Gubbins v.3.3^13^ (Croucher et al., 2015). Pairwise cgSNP differences between isolates were determined under SNP-dists v0.7.0.^14^

The resulting cgMLST allelic profile and cgSNP matrix output files, containing only the subset of genes or SNPs present in all isolates, were used by ReporTree v.2.1.2^15^ to: (i) identify potential genetic clusters based on the generated MSTreeV2, with the number of shared cgMLST alleles; (ii) conduct a phylogenetic analysis, using unique profiles with a hierarchical single-linkage clustering criterion. Finally, the phylogenetic tree was constructed by using the Maximum Likelihood method and Jukes-Cantor model in FastTree v2.1.11, incorporating 1,000 random bootstrap replicates to assess node support within the tree (Price et al., 2010). All constructed trees were exported to GrapeTree v1.5.0^16^ for visualization (Zhou et al., 2018). The threshold of 0.0035 dissimilarity (Kluytmans-van den Bergh et al., 2016), was applied for inferring genetic relatedness among *Enterobacter* spp. isolates. For cgMLST analysis, we considered ≤11 allelic differences, related; 12 to 20 allelic differences (including), possibly related; and >20 allelic differences, unrelated (Hoffmann et al., 2023). For the cgSNP phylogenetic tree, we considered a group of isolates to form a clade if they shared a common ancestor and were supported by a bootstrap value of at least 95%.

### 2.4 Data availability

The genomes of the 61 bacterial isolates included in this study were deposited in GenBank under BioProject number PRJNA1142223. More information regarding accession numbers, contigs, consensus length and average coverage is available in Supplementary Table S1. New alleles numbering for *β*-lactamases-encoding genes were requested at NCBI^17^ and are the following:*bla*_ACT-125_ (OR880573), *bla*_ACT-126_ (OR880574), *bla*_ACT-127_ (OR880575), *bla*_ACT-128_ (OR880576), *bla*_ACT-129_ (OR880577), *bla*_ACT-130_ (OR880578), *bla*_ACT-131_ (OR880579), *bla*_ACT-132_ (OR880580), *bla*_ACT-133_ (OR880581), *bla*_ACT-134_ (OR880582), *bla*_ACT-135_ (OR880583), *bla*_ACT-136_ (OR880584), *bla*_ACT-137_ (OR880585), *bla*_ACT-138_ (OR880586), *bla*_ACT-139_ (OR880587) and *bla*_MIR-26_ (OR880572).

## 3 Results

### 3.1 Diversity of *Enterobacter* spp. in environmental compartments and antibiotic susceptibility

**Figure 2:**
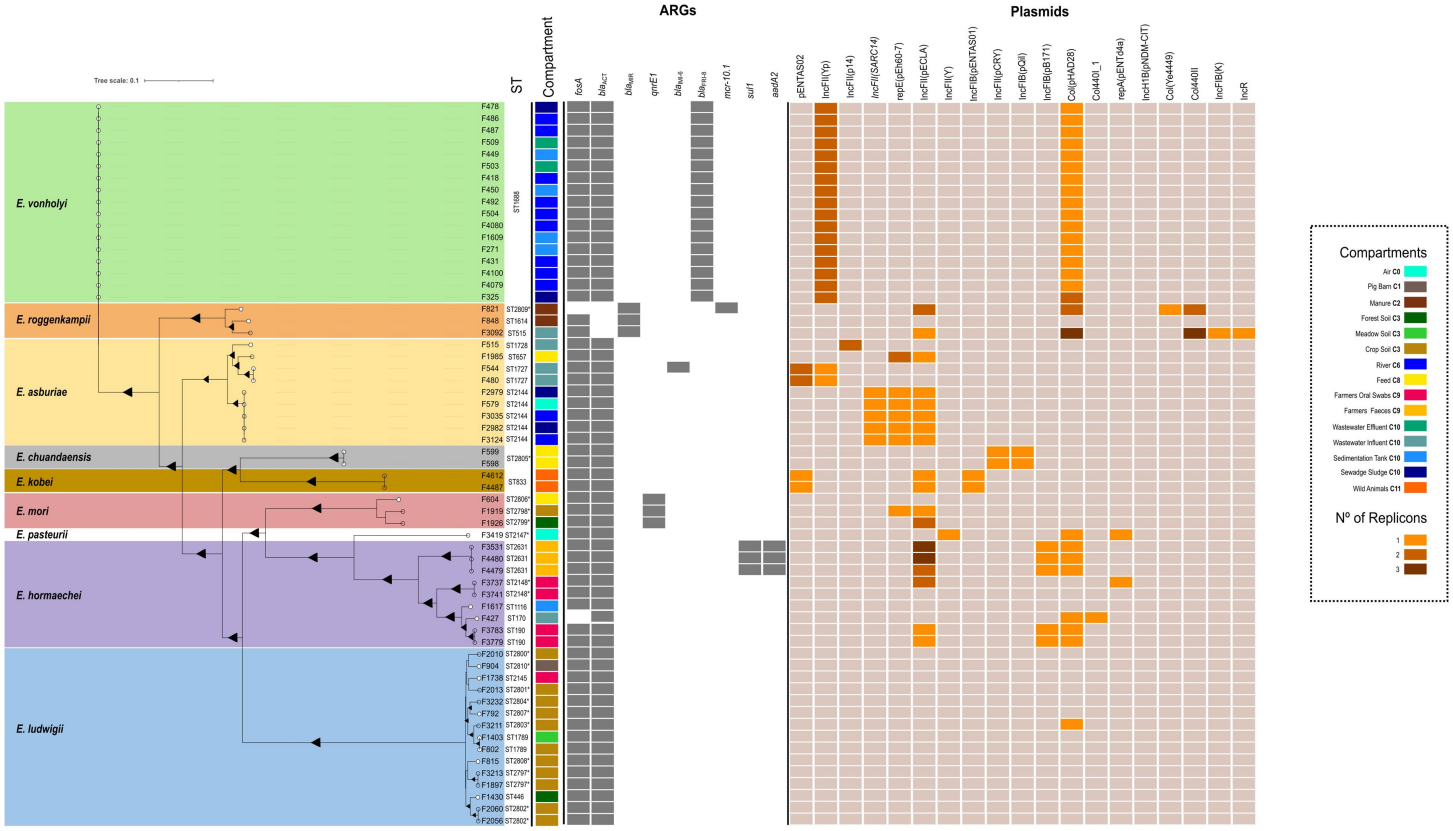
Core genome SNP (cgSNP)-based phylogenetic tree constructed with the maximum-likelihood method based on cgSNP (202,166 SNPs) alignment of 61 *Enterobacter* spp. isolates, using the *E. cloacae* 1,382 complete genome (NZ_OW968328.1) as a reference. The tree is drawn to scale, with branch lengths measured in the number of substitutions per site, and with the representation of the 7-gene MLST after the designation of each isolate. *New STs identified in this study. Isolates derived from different environmental compartments are depicted in different colors. ARGs are denoted by gray-filled squares for presence and empty squares for absence, while the number of plasmid replicons of each isolate is expressed by a color gradient (1–3). Dark triangles at branch points indicate bootstraps percentages greater than 80%. Tip color of each isolate indicate sampling year: white represents isolates from 2020, while transparent (no color) denotes isolates from 2021.

Over the annual longitudinal study covering a crop growing period, we identified 61 *Enterobacter* isolates recovered from 9 out of 12 compartments within human [farmers feces and oral swabs (C9)], animal [pig (C1) and wild animals (C11) feces], plant-associated [feed (C8)], and environmental [air of pig barns (C0), manure (C2), soil (C3) and water (C6 and C10)] reservoirs. The majority of the collection was obtained from wastewater C10 (17/61), soil C3 (15/61) and river water C6 (10/61) (Figure 1; Supplementary Figure S2; Supplementary Table S2). Taxonomic affiliation of these isolates was distributed among nine different *Enterobacter* species, with *E. vonholyi* (17/61) and *E. ludwigii* (15/61) being the most predominant (Figure 2; Supplementary Table S2). ANI analysis clarified the affiliation of the *E. vonholyi* isolates, previously identified as *E. cloacae* by the VITEK® MS mass spectrometer (Supplementary Table S2). Two different *E. hormaechei* subspecies were also identified, namely *E. hormaechei* subsp. *hoffmannii* and *xiangfangensis*. MLST analysis of the 61 isolates revealed notable diversity, identifying 32 different STs, including 16 newly assigned ones (Figure 2; Supplementary Table S2; Supplementary Figure S3). ST1688 was consistently assigned to all *E. vonholyi* isolates, while ST833 was associated with all *E. kobei* isolates.

Most *Enterobacter* isolates exhibited resistance to both amoxicillin-clavulanic acid (59/61) and cefoxitin (57/61) (Supplementary Table S2), which is an expected resistant phenotype.^18^ In contrast, all isolates were susceptible to fluoroquinolones (ciprofloxacin and enrofloxacin), trimethoprim/sulfamethoxazole, aminoglycosides (gentamicin), chloramphenicol and most of the β-lactams tested, including cefotaxime, cefepime, piperacillin/tazobactam and ceftiofur. Species-specific trends in susceptibility were also observed. Notably, all *E. chuandaensis* (*n* = 2), and *E. vonholyi* (*n* = 17) isolates, along with most of the *E. asburiae* isolates (8/9) were resistant to colistin, as well as two *E. roggenkampii* isolates and one *E. kobei* isolate, accounting for nearly half of the *Enterobacter* collection (30/61). Furthermore, all *E. vonholyi* isolates showed resistance to ertapenem and resistance or reduced susceptible to meropenem and imipenem (Supplementary Table S2). Additionally, *E. asburiae* isolate F544 exhibited resistance to all tested carbapenems while isolate F3124 displayed resistance to aztreonam, ceftazidime and ertapenem.

### 3.2 Phylogenetic and core-genome analysis

**Figure 3:**
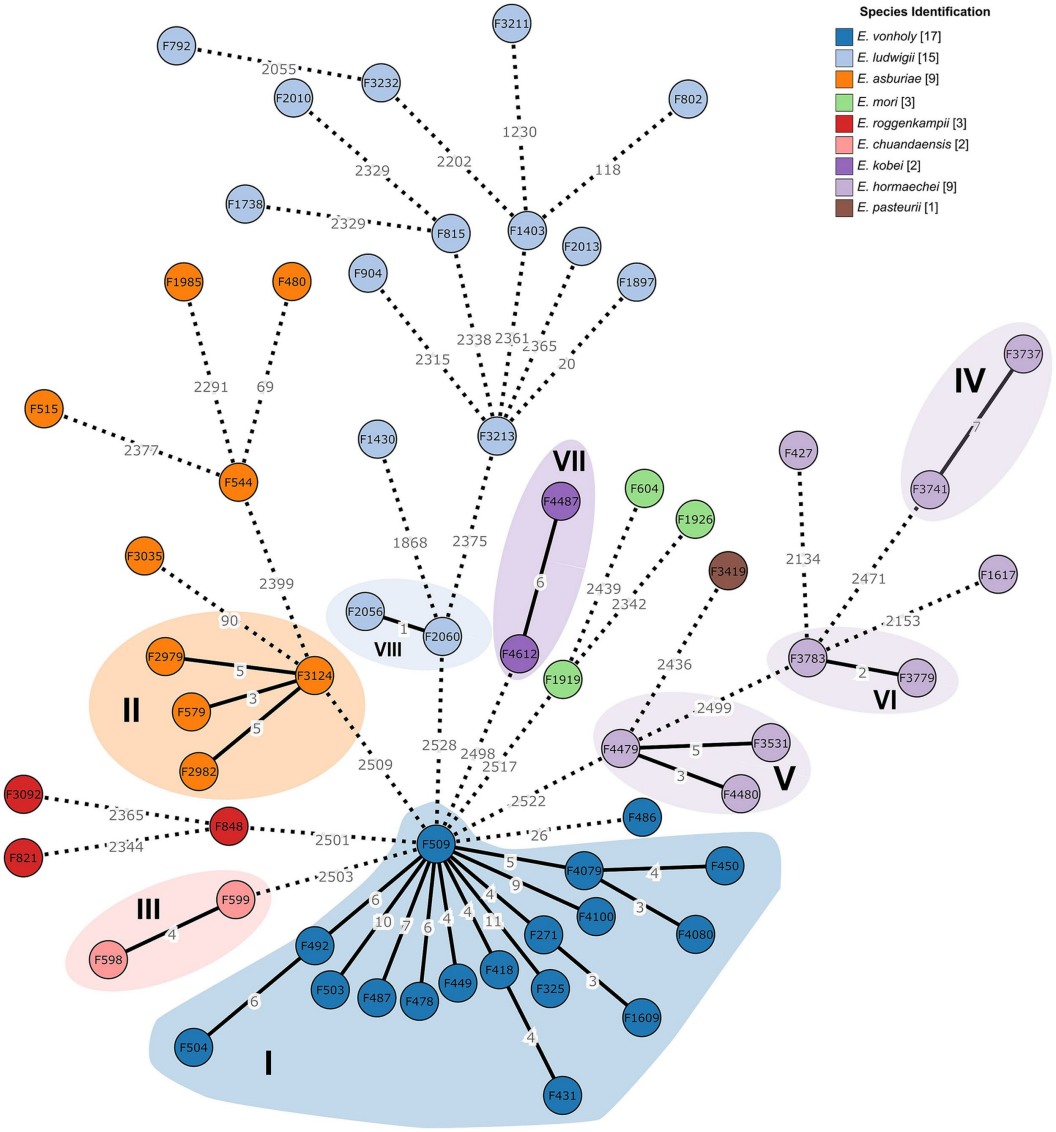
Minimum spanning tree of *Enterobacter* spp. isolates (*n* = 61), colored by specie, showing the identified clusters (from I to VIII in roman numerals) constructed based on the cgMLST analysis of 2,659 core genes. The numerical values assigned to the branches indicate the allelic distance between the isolates. Solid lines represent a distance of ≤ 11 loci (closely related). Branches longer than 20 alleles different were shortened and are indicated with a hashed line.

cgMLST analysis identified 2,534 core genes that were present in at least 95% of the 61 *Enterobacter* spp. genomes, revealing eight clusters characterized by isolates genetically related with 11 or less alleles in difference (Figure 3; Supplementary Figures S2–S4).

Cluster I encompassed all ST1688 *E. vonholyi* isolates, except F486. They exhibited a maximum allelic difference of 11 loci when each isolate within the cluster is directly compared. This cluster included only water-associated isolates, derived from wastewater ([C10] effluent, sedimentation tank, and sewage sludge) and river samples (C6), collected in two different seasons.

Cluster II consisted of four *E. asburiae* belonging to ST2144, exhibiting a maximum allelic difference of 10 loci within the cluster. These isolates were obtained from different environmental compartments, specifically air and wastewater (including sedimentation tank and sewage sludge) collected in different seasons during a one-year crop-growing period. Cluster III included the two *E. chuandensis* isolates (four loci difference), belonging to ST2805 and isolated from feed samples.

Cluster IV contained the isolate F3741 *E. hormaechei* subsp. *xiangfangensis* and *E. hormaechei* F3737, both belonging to ST2148 (seven loci difference). Cluster V comprised all three ST2631 *E. hormaechei* subsp. *hoffmannii* isolates, with a maximum allelic difference across the cluster of eight loci, while Cluster VI encompassed the two ST90 *E. hormaechei* subsp. *xiangfangensis* isolates (two locus difference). All *E. hormaechei* isolates comprised in Clusters IV, V and VI were obtained from the farmer’s oral swabs or feces. Two cgMLST clusters of *E. kobei* (Cluster VII) and *E. ludwigii* (Cluster VIII) isolates were observed (two isolates in each cluster), with a maximum allelic difference across the clusters of six and one loci, respectively. *E. ludwigii* F1897 and F3213 isolates, obtained from distinct soil samples, were considered possibly related, exhibiting 20 allelic differences. Specifically, isolate F1973 was collected from soil without organic fertilization sampled 4 weeks after corn cultivation while isolate F3212 was collected from soil with organic fertilization collected before corn harvest.

The maximum likelihood phylogenetic tree grouped isolates into nine clades, each representing different *Enterobacter* species (Figure 2). cgSNP-calling analysis resulted in an alignment of 202,166 cgSNPs, with 46.8% average of core genome alignment. The cgSNP matrix (Supplementary Table S3) displays the pairwise SNP distances among the isolates’ genomes, ranging from a minimum of 1 to a maximum of 85,835 SNPs. cgMLST identified closely related *E. vonholyi* isolates in Cluster I, with 3-11 allelic differences, which was further supported by the 1-11 cgSNP variation. The *E. vonholyi* F486 differed from other *E. vonholyi* isolates by 11-18 SNPs, classifying it as possibly related.

Overall, *Enterobacter* isolates within cgMLST Clusters II and III exhibited an average difference of 6 and 16 SNPs, respectively, whereas *E. hormaechei* isolates across Clusters IV, V and VI displayed an average difference of 31 to 41 SNPs within their clusters (Supplementary Table S3). Isolates from clusters VII and VIII showed 33 and 14 cgSNPs difference, respectively while the *E. ludwigii* isolates F1897 and F3213 differed by 8 SNPs (Supplementary Table S3).

Our phylogenetic analysis of *Enterobacter* spp. employed both cgMLST and cgSNP. The cgMLST analysis identified 2,534 core genes present in at least 95% of the 61 *Enterobacter* spp. genomes, comparable to the cgMLST schemes for *E. coli* (2,513 loci) and several *Klebsiella* spp. (2,536 loci) (Jolley et al., 2018; Zhou et al., 2020). Complementing this, the cgSNP analysis revealed a 46.8% core-genome alignment across nine different *Enterobacter* species. This percentage represents a significant proportion of shared genomic content at the genus level, particularly when compared to studies on other genera such as *Pseudomonas*, which found a very narrow core genome comprising only 65 genes out of a total of 19,056,667 coding sequences analyzed across 3,274 genomes (Saati-Santamaría et al., 2022).

While species-specific cut-offs for cgMLST and cgSNP are unlikely to be universally applicable, we utilized both approaches to provide a comprehensive view of genomic relationships within this diverse genus (Schürch et al., 2018).

### 3.3 Prevalence and distribution of ARGs, virulence factor-encoding genes and plasmids

The genomic analysis of *Enterobacter* spp. isolates revealed 29 different ARGs, conferring resistance to six antibiotic classes (Figure 2; Supplementary Table S4). All *Enterobacter* isolates exhibited a common resistome marked by the expression of a chromosomally encoded *ampC*-type gene. Indeed, a *bla*_ACT_ gene was identified in all species except in *E. roggenkampii* isolates, which carried a *bla*_MIR_ gene instead. Fourteen new *bla*_ACT_ variants and one novel *bla*_MIR_ variant were identified (Supplementary Table S4). Furthermore, except for *E. roggenkampii* F821 and *E. hormaechei* subsp. *hoffmannii* F427, all isolates harbored a chromosomally encoded *fosA*-like gene that conferred resistance to fosfomycin. Notably, two carbapenemase-encoding genes (*bla*_FRI-8_ and *bla*_IMI-6_) were identified, along with resistance genes for colistin (*mcr-10.1*), quinolone (*qnrE1*), sulfonamide (*sul1*) and streptomycin (*aadA2*). *bla*_FRI-8_ was present in all *E. vonholyi* isolates, *bla*_IMI-6_ in one *E. asburiae* ST1727 isolate, *mcr-10.1* in *E. roggenkampii* ST2809 and *qnrE1* in three *E. mori* isolates, while both *sul1* and *aadA2* genes were present in the three *E. hormaechei* subsp. *hoffmannii* isolates (Figure 2). Additionally, most isolates exhibited membrane-associated resistance mechanisms, including efflux pumps and reduced outer-membrane permeability (Supplementary Table S4).

The distribution of virulence factor-encoding genes (VFs), plasmid replicons, as well as the presence of metal, heat and biocides resistance-encoding genes were also investigated (Supplementary Table S4). The *fieF* gene, associated with flagellum biosynthesis, was detected in all *Enterobacter* isolates. Additionally, siderophore-encoding genes (*iroB*, *iroC*, *iroN*) were found in four *E. hormaechei* subsp. *xiangfangensis* (Supplementary Table S4). Gene clusters encoding resistance to arsenic, cobalt, nickel, copper, silver and tellurite were present in isolates from six different *Enterobacter* species. Moreover, a locus associated with heat shock response was detected in all three *E. hormaechei* subsp. *hoffmannii* isolates and in one *E. roggenkampii* (Supplementary Table S4). Most *Enterobacter* species harbored at least two different plasmid replicons, except for *E. ludwigii*, for which replicons were only detected in one isolate (Figure 2). Additionally, the same plasmid profile was detected in all *E. vonholyi* isolates, consisting of two IncFII(Yp) replicons and a Col(pHAD28) (Figure 2).

### 3.4 Plasmid analysis and genomic context of ARGs

**Figure 4:**
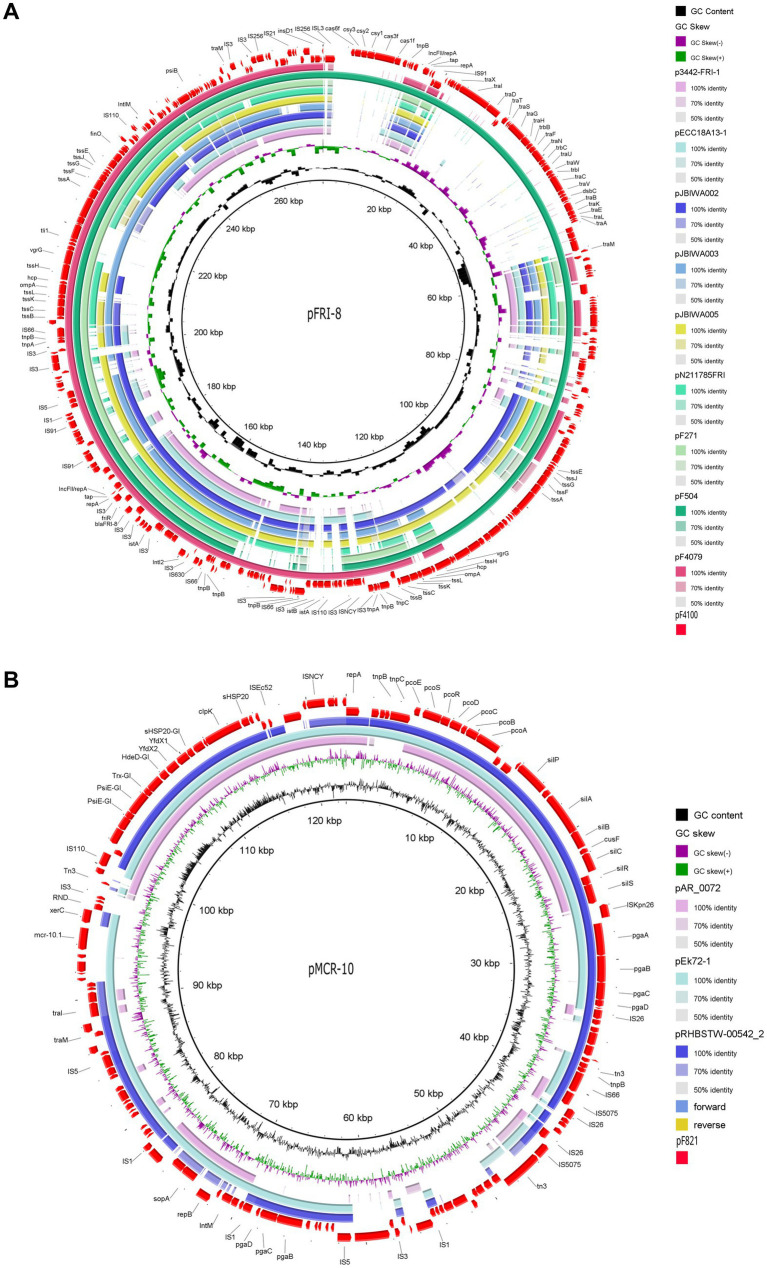
Mapping and circular comparison of *bla*_FRI-8_- **(A)** and *mcr-10* **(B)** carrying plasmid sequences displaying the genomic location of ARGs, MGEs, VFs, genes associated with plasmid conjugation and also heat and metal resistance genes. The pF4100 and pF821 plasmids were used as reference for the *bla*_FRI-8_ and *mcr-10.1* comparisons, respectively. The ring color gradients correspond to varying degrees of identity of BLAST matches. Circular genomic maps also include information on GC Skew and GC content. Plasmids used for sequence comparison are the closest plasmid sequences obtained using NCBI BLAST analysis.

Hybrid genome assembly analysis of four *bla*_FRI-8_ positive *Enterobacter* isolates (F271, F504, F4079 and F4100) identified four IncFII(Yp) plasmids, the plasmids pF271 (PQ133128), pF504 (PQ133129), pF4079 (PQ133130) and pF4100 (PQ133131), ranging from 120 to 272 to kb in size (Figure 4A). Beforehand, short-read sequencing analysis strongly indicated that in all the remaining *E. vonholyi* isolates, the *bla*_FRI-8_ gene was located on IncFII(Yp) plasmids in regions identical to pF271. While F504, F4079, F4080 and F4100 exhibited unique sequences absent from pF271, the plasmids in F4080 and F4100 showed such a high degree of similarity that they were considered potentially identical. Furthermore, pF271 exhibits a 99.9% nucleotide sequence similarity to pF4079, excluding the 21 kb segment present in pF4079 but absent from pF271 approximately in the region between 132 and 152 kb (Figure 4A), which harbors several MGEs including insertion sequences and transposase coding genes.

**Figure 5:**
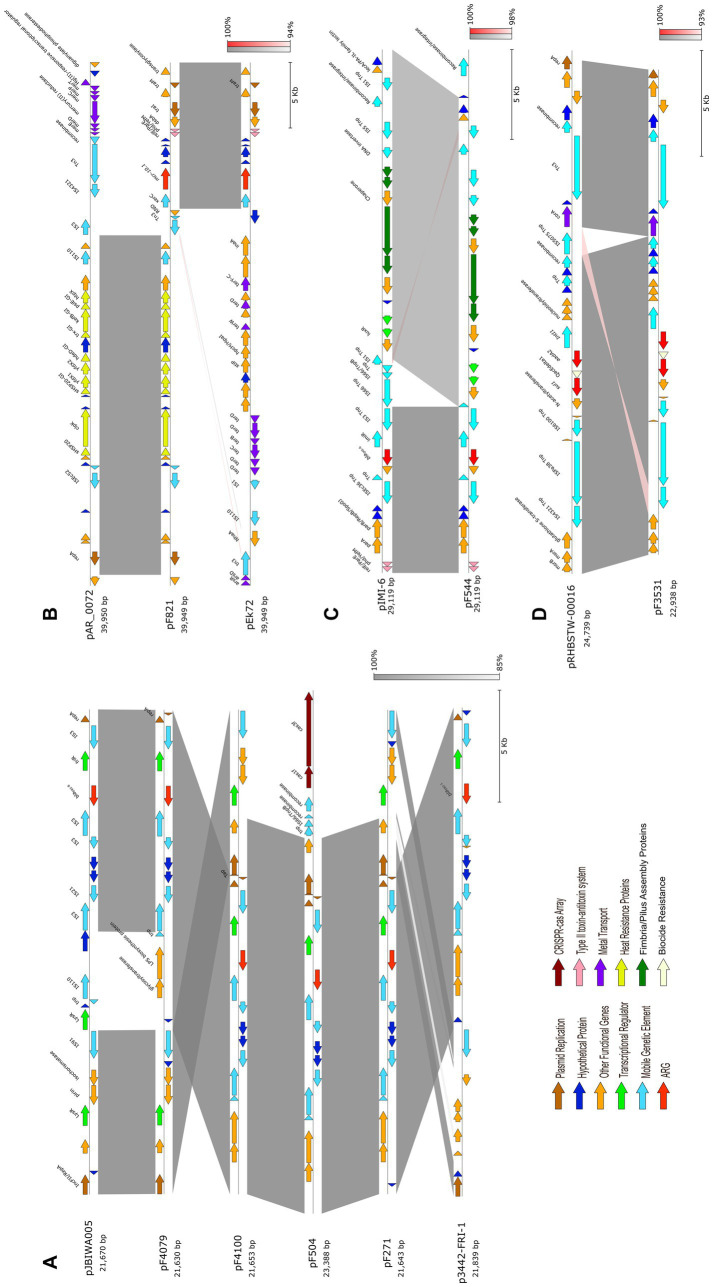
Genetic environment and comparative linear analysis of *bla*_FRI-8_ **(A)**, *mcr-10* **(B)**, *bla*_IMI-6_ **(C)** and the class 1 integron **(D)** identified in *Enterobacter* isolates performed and visualized with pyGenomeViz. pJBIWA005_1 (CP074160) and p3442-FRI-1 (CP033467) were included in the *bla*_FRI-8_ comparison, pAR_0072 and pEk72 for *mcr-10.1*, pIMI-6 (KX786187) for *bla*_IMI-6_ and pRHBSTW-00016_2 (CP058188) for the class 1 integron. Arrows indicate the directions of transcription of the genes, and different genes are shown in different colors according to their function (detailed in the legend). Regions of homology are depicted using gray and red gradients, with red signalizing inversions. Arrows are drawn to scale.

Despite size and structure differences among plasmids carrying *bla*_FRI-8_, the region around this carbapenemase-encoding gene is highly conserved (Figure 5A). In each plasmid, *bla*_FRI-8_ and the transcription regulator (*friR*) were flanked upstream and downstream by *IS*3 family transposase-encoding genes (Figures 4A, 5A). Additionally, proteins associated with type VI secretion systems were detected in all four plasmids.

The pF504 and pF4100 are larger plasmids with a high degree of similarity to each other but show less resemblance to other *bla*_FRI_-harboring plasmids. These two plasmids shared a CRISPR (clustered regularly interspaced short palindromic repeats)-Cas (CRISPR associated proteins) system, although located in a different genetic context (Figure 5A).

Hybrid genome assembly analysis of isolate F821 identified a 122 kb IncFII(pECLA) plasmid pF821 (PQ133132) harboring the *mcr-10.1* gene (Figure 4B). The *xerC* recombinase is located downstream a gene encoding an efflux RND transporter permease subunit and two transposases (Figures 4B, 5B). In addition to several genes encoding MGEs, pF821 also encompasses genes encoding for heat-shock proteins identically to the pAR_0072 plasmid (Figures 4B, 5B), but in this case located upstream the *xerC* - *mcr-10.1* region. Furthermore, it contains a *pgaABCD* operon, which is responsible for the synthesis, modification and export of poly-*β*-1,6-*N*-acetyl-D-glucosamine, an adhesin crucial for biofilm formation (Itoh et al., 2008). Additionally, pF821 encompasses two operon systems conferring resistance to copper and silver (Figure 4B).

Short-read sequencing analysis of F544 revealed that both the *bla*_IMI-6_ and the transcription regulator gene *imiR* were flanked by two *IS*3 family transposases (Figure 5C). In *E. hormaechei* subsp. *hoffmannii* isolates F3531, F4479 and F4480, the analysis of the genetic context of an *aadA2* gene revealed its location in a class 1 integron with the respective structural genes of this class, *sul1* and *qacEdelta1* (Figure 5D). Moreover, the contig containing the 5’CS: *IntI1*|*aadA2*|*qacEdelta1*|*sul1* gene cassette exhibited 100% nucleotide sequence similarity with pRHBSTW-00016 plasmid (CP058188.1), which was also described in an *E. hormaechei* isolate.

## 4 Discussion

In this work, we explored the genomic epidemiology and AMR genetic portfolio of *Enterobacter* isolates obtained from a Portuguese OAL, emphasizing on AMR dynamics across interconnected environmental compartments. The collection of *Enterobacter* isolates underscores the complexity of this genus, highlighting its diversity through the identification of numerous different species (*n* = 9) and previously unrecognized STs (*n* = 16) (Davin-Regli et al., 2019). The nine different compartments including environment [water (C6 and C10), soil (C3), manure (C2), and air (C0)], human (C9) and animal [(C1, C8, and C11)] from which these isolates were recovered also reflect the ubiquity of *Enterobacter* species. In fact, these organisms are commonly found in diverse environments and can also act as commensals and opportunistic pathogens, causing nosocomial and community-acquired infections (Gomi et al., 2018; Brouwer et al., 2019; Liao et al., 2022; Manageiro et al., 2022; Furuichi et al., 2024). Moreover, investigating diverse compartments within the One Health framework provides a holistic view of AMR transmission routes and can help uncover the complex pathways through which ARGs disseminate.

Animal production farms, like our simulated OAL area, have been recognized as significant reservoirs of AMR, harboring bacteria resistant to last-resort antibiotics such as carbapenems and colistin (Wang et al., 2017; Yang et al., 2022).

The global increase in environmental *Enterobacterales* resistant to these antibiotics underscores the urgent need to comprehend emerging resistance mechanisms. Thus, investigating less common or emerging ARGs is critical for anticipating new potential AMR threats.

The identification of a diverse ARGs alongside MGEs in our samples reinforces the role of these species as vectors for AMR transmission and dissemination (De Oliveira et al., 2020). The co-occurrence of antibiotic and metal resistance genes on the same bacterial isolates is expected, as these genes are often governed by shared genetic mechanisms and can be encoded by the same MGEs (Roberto et al., 2019). Furthermore, these environments are frequently contaminated with metals, which can lead to co-selection of resistant isolates (Silva et al., 2021). Notably, this study has documented new ARG variants (*bla*_ACT-25_ to *bla*_ACT-39_ and *bla*_MIR-26_) and highlighted the prevalence of ARGs conferring resistance to last resort antibiotics within *Enterobacter* species, including *mcr* and carbapenemase-coding genes, thereby supporting and complementing previous findings (Peirano et al., 2018; Manageiro et al., 2022; Teixeira et al., 2022).

FRI class A carbapenemases were first described in an *E. cloacae* strain isolated from a hospitalized patient in Paris, sharing closest amino acid identity with chromosome-encoded Ambler class A carbapenemases NMC-A and IMI-1 (Dortet et al., 2015). FRI-1 significantly hydrolyzes carbapenems, conferring resistance to aztreonam, but not to broad-spectrum cephalosporins (Dortet et al., 2015). This phenotype aligns with previous reports of *bla*_FRI-8_-harboring *Enterobacter* isolates and matches the resistance profile of most *bla*_FRI-8_-harboring *E. vonholyi* isolates described here.

Currently, 12 different *bla*_FRI_ variants have been described, with all except *bla*_FRI-10_ (found in *E. coli*) detected in *Enterobacter* species. Additionally, *Enterobacter* isolates with *bla*_FRI_ have been detected in both environmental and clinical settings, indicating potential transmission events (Gomi et al., 2022; Mataseje et al., 2023). In Europe, *bla*_FRI_ has exclusively been reported in clinical *Enterobacter* strains, although most reports come from Asian countries (Dortet et al., 2015; Meunier et al., 2017; Schauer et al., 2019; Gomi et al., 2022; Wu et al., 2023). This report represents, to the best of our knowledge, the first documentation of *bla*_FRI_-harboring *Enterobacterales* in environmental settings in Europe and overall, in Portugal. The association of *bla*_FRI_ with *E. vonholyi* has been recently described by Cho et al. (2021). Prior to this, *bla*_FRI_ genes were primarily linked to various *Enterobacter* species. Notably *bla*_FRI-6_ and *bla*_FRI-8_ have been identified in *E. vonholyi* isolates previously classified as *Enterobacter* spp. (Boyd et al., 2020; Gomi et al., 2022). This reclassification underscores the importance of ongoing taxonomic revisions in understanding ARGs distribution across bacterial species.

The *bla*_FRI-8_-harboring plasmids described here share a high homology with each other and with other *bla*_FRI_-harboring plasmids described previously in *Enterobacter* isolates (Brouwer et al., 2019; Adachi et al., 2021; Gomi et al., 2022; Mataseje et al., 2023) (Figure 4A). Notably, plasmids pF271 and pF4079 exhibit approximately 99% sequence similarities with 87 to 90% of the pJBIWA003 nucleotide sequence, a plasmid identified in an *E. quasiroggenkampii* isolate recovered from surface water in Japan (Gomi et al., 2022).

Previous work has shown that *bla*_FRI_-carrying plasmids are not self-transmissible, except for *bla*_FRI-6_, also detected in *E. vonholyi* (Dortet et al., 2015; Kubota et al., 2018; Schauer et al., 2019; Uwamino et al., 2019; Boyd et al., 2020). Analysis of the *bla*_FRI-8_ associated plasmids sequences described here unveiled that only pF504 and pF4100 harbor a conjugation module comprising *tra* and *trb* genes (Figure 4A). This module shares the same overall gene structure as the one the present in the self-transmissible *bla*_FRI-6_-carrying plasmid (CP034768), potentially enablingpF504 and pF4100 be transferred between hosts (Boyd et al., 2020; Wu et al., 2023). The incomplete conjugation module in most *bla*_FRI_-carrying plasmids might explain their low prevalence and nearly exclusive association with *Enterobacter* species. Nonetheless, the abundance of other MGE within these plasmids, particularly transposase-coding genes and insertion sequences, could eventually potentiate the transfer of both *bla*_FRI_ and the transcriptional regulator *friR* to highly transmissible plasmids, potentially facilitating their widespread dissemination.

Along with FRI, IMI *β*-lactamases are classified as “minor” carbapenemases, sporadically described across different continents (Bonnin et al., 2021). IMI-1 was initially reported in the USA in an *E. cloacae* strain and to date 24 different variants have been described, mostly in *Enterobacter* species (Rasmussen et al., 1996). IMI-6, first identified on an IncFII-type plasmid originating from a clinical *E. cloacae* isolate from Canada, and has since been exclusively detected in these species (Boyd et al., 2017; Blanco-Martín et al., 2023). Similar to *bla*_FRI-8_, this represents the first description of *bla*_IMI_ in Portugal. The genomic context containing these genes shares 99.2% nucleotide identity with the corresponding region of the pIMI-6 plasmid from the ST283 *E. asburiae* clinical isolate obtained from a Canadian hospital, suggesting that this gene is likely present within a pIMI-6-like plasmid (Boyd et al., 2017). In addition to the high nucleotide identity, the presence of genes associated with pilus biogenesis in the same region, as well as genes associated with copper resistance and an IncFII replicon further supports this possibility (Boyd et al., 2017). Furthermore, *E. asburiae* isolate F544 exhibited resistance to carbapenems, but not to extended-spectrum cephalosporins, consistent with previously descriptions of *Enterobacter* isolates harboring *bla*_IMI_ (Sugawara et al., 2019; Blanco-Martín et al., 2023).

Colistin-resistance poses an emerging threat to public and environmental health. Among 10 currently described *mcr* variants, *mcr-1* exhibits the highest prevalence, particularly in *E. coli* (Zhang et al., 2021; Hu et al., 2023). *mcr-10* was first described in 2020, detected on a IncFIA plasmid of a clinical *E. roggenkampii* isolate in China (Wang et al., 2020). Since then, *mcr-10* has been identified in various *Enterobacterales* species in many countries, indicating its widespread dissemination (Biggel et al., 2022; Xu et al., 2022). In Portugal, *mcr-1*-harboring *Enterobacterales* have been extensively reported across various matrices, including clinical settings (Beyrouthy et al., 2017; Tacão et al., 2017), livestock (Clemente et al., 2019; Manageiro et al., 2019; Palmeira et al., 2021; Ribeiro et al., 2021), wild animals (Ahlstrom et al., 2019; Torres et al., 2021; Dantas Palmeira et al., 2022) and vegetables (Manageiro et al., 2020). In Portugal, besides *mcr-1*, two other variants have been reported; *mcr-9* gene has been detected in diverse settings: in an *E. ludwigii* isolate recovered from a fish farm, an environmental *Klebsiella quasipneumoniae* isolate, and in *Salmonella enterica* serovar Typhimurium and its monophasic variant clinical isolates (Manageiro et al., 2022; Silveira and Pista, 2023; Silva et al., 2024). Additionally, *mcr-4* has been detected on *E. coli* isolates recovered from pigs (Amaro et al., 2023). Hence, to the extent of our knowledge, this corresponds to the first publication of *mcr-10* in Portugal. The hybrid genome assembly analysis of pF821 plasmid revealed high similarity with other IncFIB(pECLA) plasmids also previously identified in *Enterobacter* species, although only pEk72-1 (CP088230) harbored *mcr-10.1* (Figure 4B). On both plasmids, a tyrosine-type recombinase gene *xerC* was located upstream of *mcr-10.1* (Figures 4B, 5B). The region encompassing both *xerC* and *mcr-10.1*, along with the downstream segment, exhibits a 99.9% nucleotide identity with the corresponding region of pEk72, a plasmid described in a clinical *E. kobei* strain isolated in a Chinese hospital (CP088230). The occurrence of *mcr-10* in association with a *xerC* tyrosine recombinase and in close proximity to diverse ISs, reinforces previous studies indicating that this structure is highly conserved and prone for the mobilization of the *mcr-10* gene (Xu et al., 2021, 2022; Yang et al., 2021). Furthermore, pF821 harbors additional genes that could confer an adaptability advantage to its host, potentially enhancing its virulence. These genes include those implicated in biofilm formation (*pgaABCD* operon) as well as resistance to heat (e.g., *psiE-GI*, *kefB-GI*, *trx-GI*) and metals (e.g., *pcoE*, *pcoS*, *pcoD*). These genetic elements are often associated with plasmids that carry ARGs (Teixeira et al., 2016; Papagiannitsis et al., 2017; Lin et al., 2020). Despite harboring the *mcr-10* gene, isolate F821 remained susceptible to colistin. This is not unusual, as *mcr* genes have been identified in colistin-susceptible *Enterobacterales* (Terveer et al., 2017; Manageiro et al., 2020, 2022; Bertelloni et al., 2022). The colistin-resistant phenotype of *Enterobacter* isolates observed in this study may be linked to the overexpression of *acrA* in the *acrAB-tolC* efflux pump, potentially in combination with decreased affinity between colistin and the outer membrane due to lipid A modification, as previously reported in *Enterobacter* species (Telke et al., 2017; Liu et al., 2021).

The occurrence of *E. hormaechei* harboring a class 1 integron with *sul1* and *aadA2* isolated from farmer’s feces highlights the need for a One Health approach in tackling AMR.

While no transmission events involving these isolates were tracked between environmental compartments, we cannot rule out this hypothesis as the transmission of antibiotic resistant bacteria between farmers, animals and farm environment has been previously documented (Wang et al., 2017). Additionally, the same MGEs harboring ARGs could also be circulating between different bacterial species and compartments. Putative clonal transmission events were observed among *E. vonholyi* isolates between the stabilization pond (C10) and the river (C6), as well as among *E. asburiae* ST2144 isolates between the stabilization pond (C10) and the air of pig barns (C0). The occurrence of two *Enterobacter* isolates (F579 and F3419) isolated from the air of pig barns (C0) highlights this often-overlooked environmental reservoir, which has been previously recognized as a hotspot of antibiotic resistant bacteria (He et al., 2020; Rossi et al., 2023).

The detection of an *mcr-10* harboring *Enterobacter* isolate isolated from pig manure (C2) aligns with recent findings in Portugal describing a high rate of *mcr* harboring *Enterobacterales* isolated from pigs (Kieffer et al., 2017; Manageiro et al., 2019; Fournier et al., 2020; Amaro et al., 2023). This is particularly concerning, as the potential use of manure in farming soil can potentiate the transmission of this *mcr* harboring *Enterobacter* to crops and water meant for human consumption.

Furthermore, this study indicates that the *bla*_FRI-8_ harboring *E. vonholyi* isolates can persist in the stabilization pond for at least a period of 6 months, from summer to winter, suggesting the existence of a persistent source of contamination. Likewise, *E. asburiae* ST2144 isolates within cluster II (Supplementary Figure S4) were also isolated in different seasons, namely winter and spring. Our observation that all *Enterobacter* isolates harboring carbapenemase encoding genes were isolated from aquatic environments also confirms the well-recognized importance of these environments in the dissemination of carbapenem resistance (Manageiro et al., 2014; Hooban et al., 2020; Teixeira et al., 2022).

## 5 Conclusion

In this study, we investigated the dynamics of AMR in a Portuguese OAL testing ground. We identified diverse *Enterobacter* species across various compartments, highlighting their role as vectors for AMR dissemination. The presence of carbapenemase-encoding genes such as *bla*_FRI-8_ and *bla*_IMI-6_, along with the emerging plasmid mediated colistin resistance such as *mcr-10.1* gene, poses significant challenges to public health. Our findings emphasize the interconnected nature of AMR, elucidating the MGE contributing to the dissemination of these concerning rare and emerging ARGs. The detection of highly similar *Enterobacter* isolates across environmental compartments suggests possible transmission events and the presence of persistent reservoirs of antibiotic resistant bacteria. This highlights how essential it is to monitor the spread and emergence of ARGs, in parallel with the development of preventive measures and interventions against AMR bacteria in different environmental compartments.

## Data Availability

The genomes of the bacterial isolates included in this study were deposited in GenBank under BioProject number PRJNA1142223.

## Footnotes

^1^https://doi.org/10.5281/zenodo.5378535; https://doi.org/10.5281/zenodo.5381388; https://doi.org/10.5281/zenodo.5376614; https://doi.org/10.5281/zenodo.5381592; https://doi.org/10.5281/zenodo.5377559; https://doi.org/10.5281/zenodo.5380213; https://doi.org/10.5281/zenodo.5380081

^2^ https://doi.org/10.5281/zenodo.5381927

^3^ https://www.inrs.fr/publications/bdd/metropol/fiche.html?refINRS=METROPOL_147

^4^ https://clsi.org/standards/products/microbiology/documents/m100/

^5^ http://www.eucast.org/clinical_breakpoints/

^6^ https://github.com/B-UMMI/INNUca

^7^ http://www.bioinformatics.babraham.ac.uk/projects/fastqc/

^8^https://www.ncbi.nlm.nih.gov/datasets/genome/?taxon=547; December 11th, 2023.

^9^ http://github.com/tseemann/abricate

^10^ https://github.com/moshi4/pyGenomeViz

^11^ https://github.com/B-UMMI/chewBBACA

^12^ https://github.com/tseemann/snippy

^13^ https://github.com/nickjcroucher/gubbins

^14^ https://github.com/tseemann/snp-dists

^15^ https://github.com/insapathogenomics/ReporTree

^16^ https://github.com/achtman-lab/GrapeTree

^17^ https://www.ncbi.nlm.nih.gov/pathogens/submit-beta-lactamase/

^18^ https://www.eucast.org/expert_rules_and_expected_phenotypes/

## Supplementary material

Supplementary Figure S1 Aerial view of the Portuguese OAL in Santarém, with the location of the pig farm (C1), manure collection tank (C2), crops (C4), waste stabilization ponds (C10), drainage water from borehole (C5), irrigation tank (C6), and the river’s water line (C6) marked in orange squares.

Supplementary Figure S2 Minimum spanning tree of *Enterobacter* spp. isolates (*n* = 61), by environmental compartment, showing the identified clusters (from I to VIII in roman numerals) constructed based on the cgMLSTanalysis of 2,659 core genes.

Supplementary Figure S3 Minimum spanning tree of *Enterobacter* spp. isolates (*n* = 61), by MLST, showing the identified clusters (from I to VIII in roman numerals) constructed based on the cgMLST analysis of 2,659 core genes.

Supplementary Figure S4 Minimum spanning tree of *Enterobacter* spp. isolates (*n* = 61), by season, showing the identified clusters (from I to VIII in roman numerals) constructed based on the cgMLST analysis of 2,659 core genes.
