# Supplementary figures and images for: Genomic epidemiology and resistome dynamics of Enterobacter species in a Portuguese Open Air Laboratory: the emergence of the FRI-8 carbapenemase
Source: Front Microbiol. 2025 Jul 31;16:1593872. doi: 10.3389/fmicb.2025.1593872 (PMC12350302; doi:10.3389/fmicb.2025.1593872)

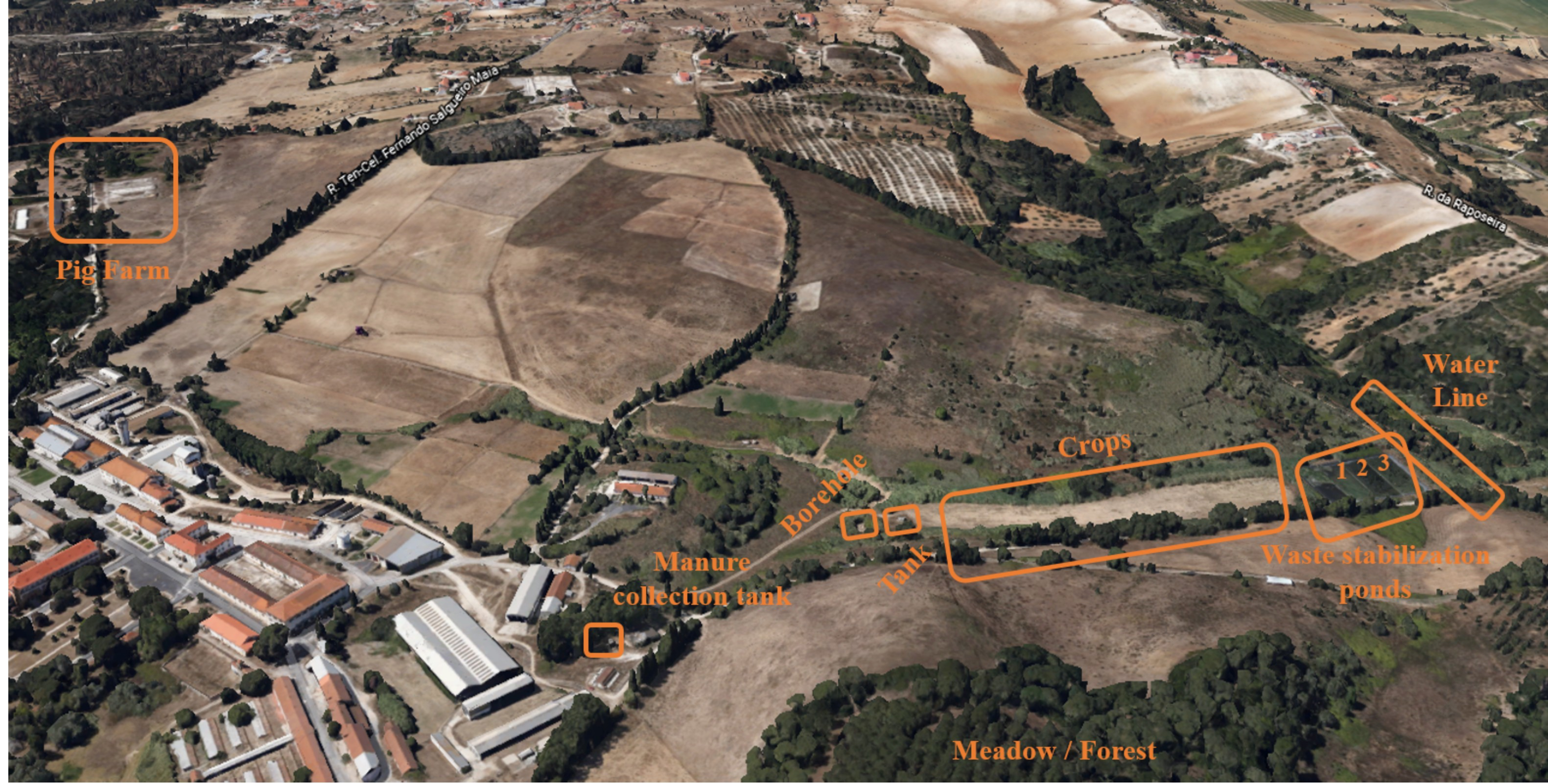

Supplement: Supplementary Figure S1 — Aerial view of the Portuguese OAL in Santarém, with the location of the pig farm (C1), manure collection tank (C2), crops (C4), waste stabilization ponds (C10), drainage water from borehole (C5), irrigation tank (C6), and the river’s water line (C6) marked in orange squares. [file Data_Sheet_1.zip › Datasheet 1/Supplementary Figure 1 .png]

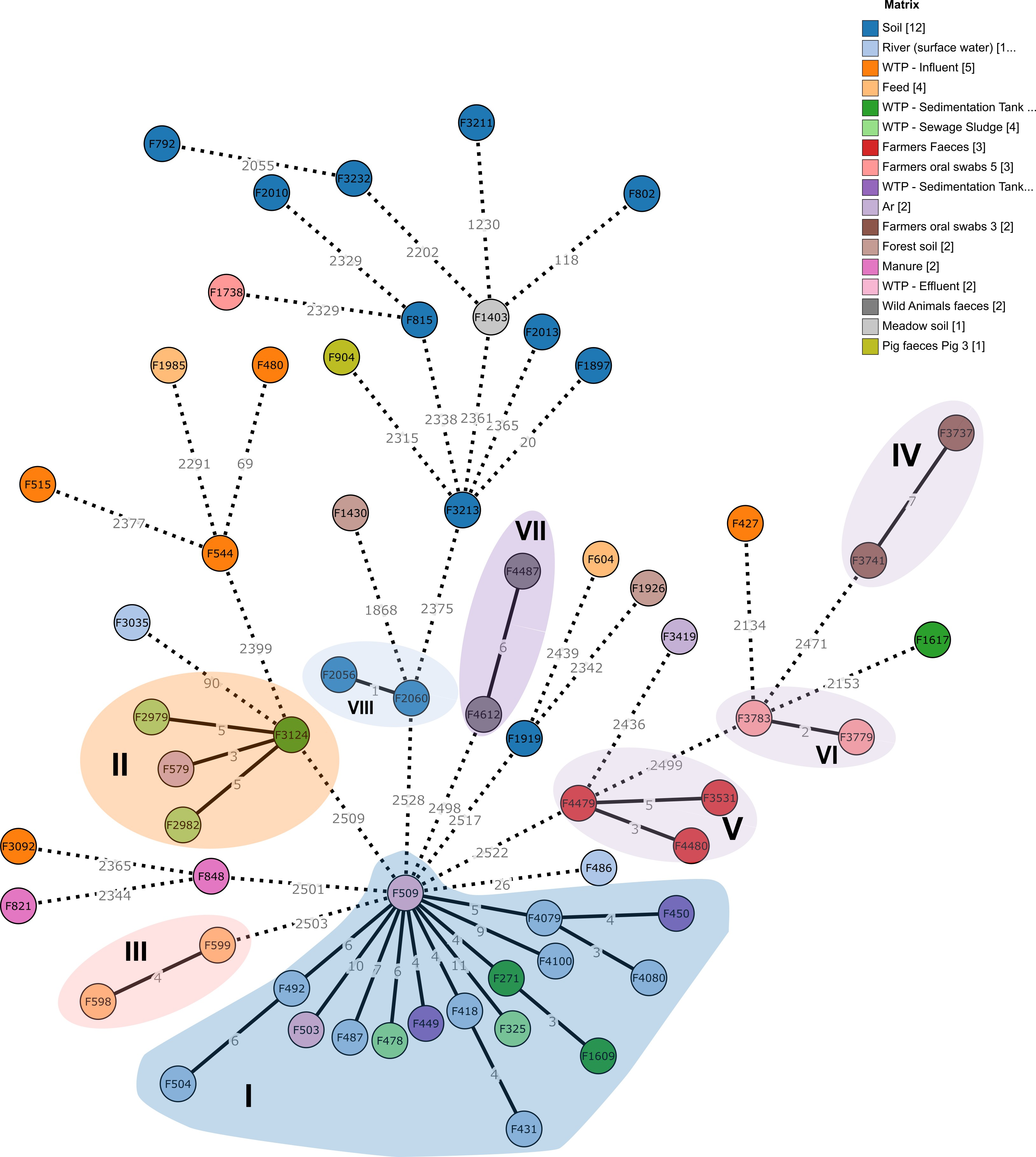

Supplement: Supplementary Figure S1 — Aerial view of the Portuguese OAL in Santarém, with the location of the pig farm (C1), manure collection tank (C2), crops (C4), waste stabilization ponds (C10), drainage water from borehole (C5), irrigation tank (C6), and the river’s water line (C6) marked in orange squares. [file Data_Sheet_1.zip › Datasheet 1/Supplementary Figure 2.png]

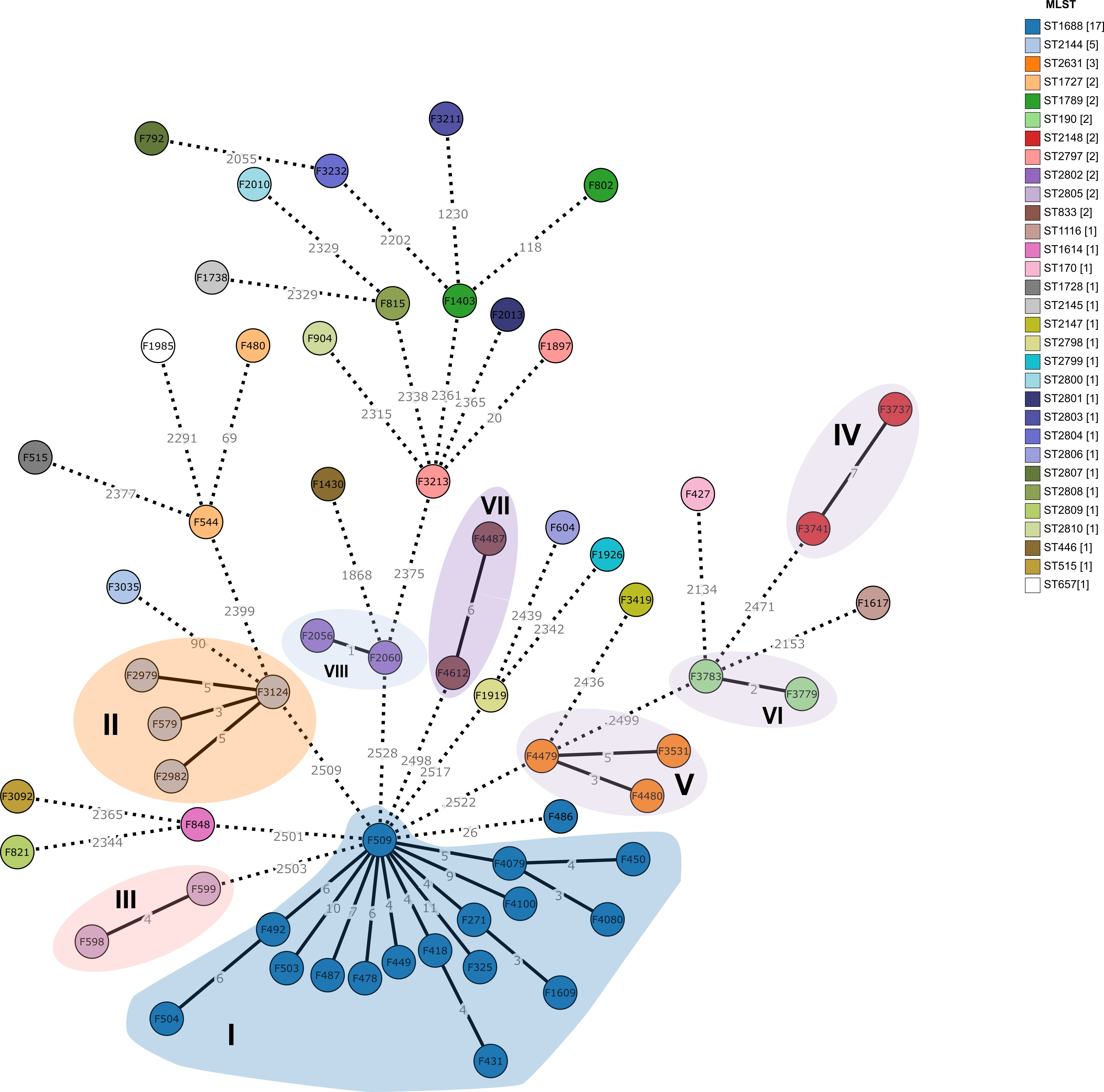

Supplement: Supplementary Figure S1 — Aerial view of the Portuguese OAL in Santarém, with the location of the pig farm (C1), manure collection tank (C2), crops (C4), waste stabilization ponds (C10), drainage water from borehole (C5), irrigation tank (C6), and the river’s water line (C6) marked in orange squares. [file Data_Sheet_1.zip › Datasheet 1/Supplementary Figure 3 .png]

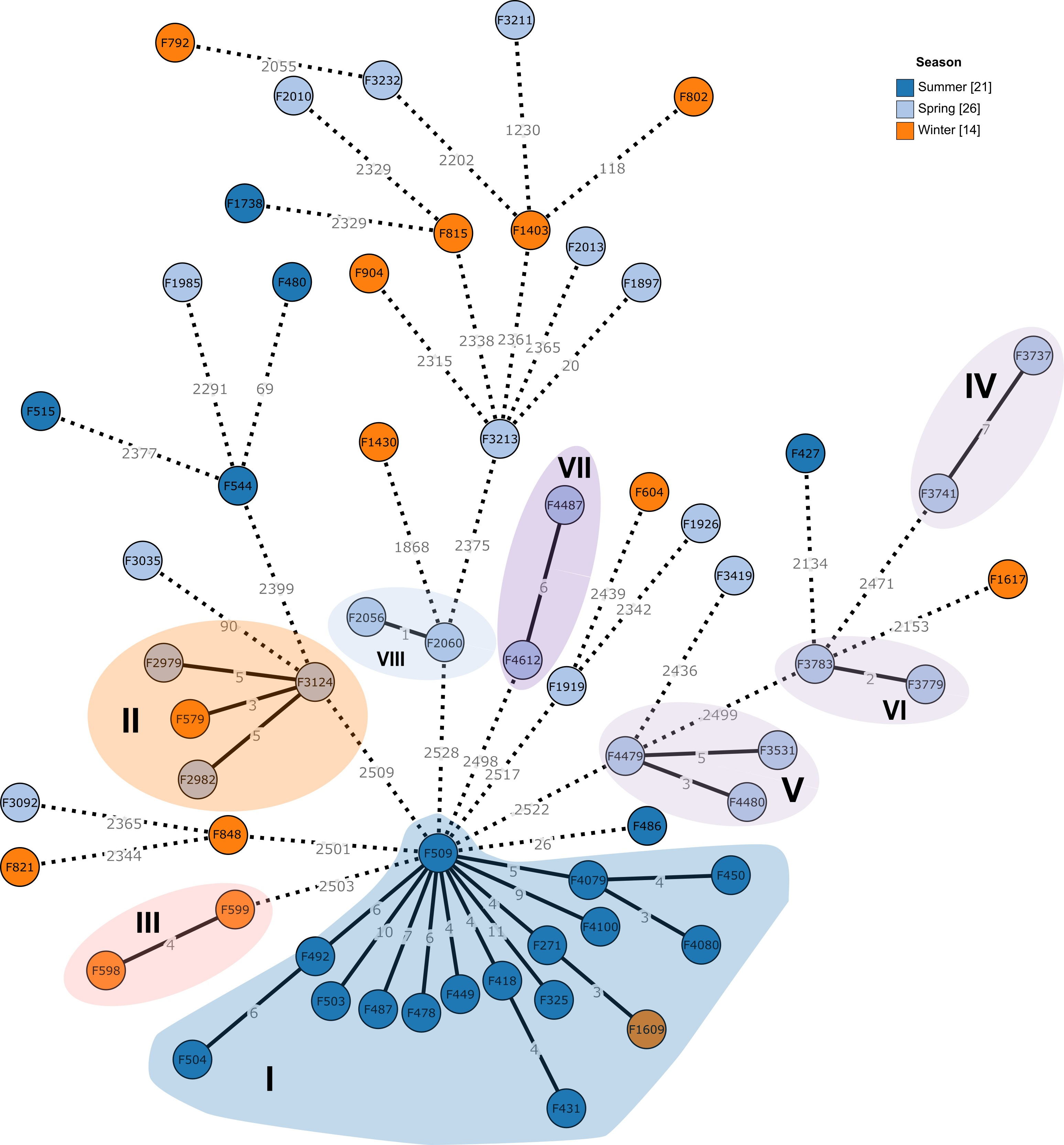

Supplement: Supplementary Figure S1 — Aerial view of the Portuguese OAL in Santarém, with the location of the pig farm (C1), manure collection tank (C2), crops (C4), waste stabilization ponds (C10), drainage water from borehole (C5), irrigation tank (C6), and the river’s water line (C6) marked in orange squares. [file Data_Sheet_1.zip › Datasheet 1/Supplementary Figure 4.png]
